# Effects of environment, dietary regime and ageing on the dengue vector microbiota: evidence of a core microbiota throughout *Aedes aegypti* lifespan

**DOI:** 10.1590/0074-02760160238

**Published:** 2016-08-25

**Authors:** Mariana Rocha David, Lilha Maria Barbosa dos Santos, Ana Carolina Paulo Vicente, Rafael Maciel-de-Freitas

**Affiliations:** 1 Fundação Oswaldo Cruz, Instituto Oswaldo Cruz, Laboratório de Mosquitos Transmissores de Hematozoários, Rio de Janeiro, RJ, Brasil; 2 Fundação Oswaldo Cruz, Instituto Oswaldo Cruz, Laboratório de Genética Molecular de Microrganismos, Rio de Janeiro, RJ, Brasil

**Keywords:** Aedes, microbiota, diversity, mark-release-recapture

## Abstract

Mosquito midgut microbiota is a key component of vector competence, as gut bacteria can disturb pathogen development. In this study, we addressed the microbiota composition of *Aedes aegypti* during its lifespan, under field conditions. We also investigated the possible effects of environment, dietary regime and ageing on the gut community composition. We employed culture independent and dependent approaches to characterise vector microbiota. There was evidence of a lifelong stable core microbiota after mosquitoes were released into an urban settlement, where they presumably fed on a range of vertebrate hosts and carbohydrate sources. This core was formed mainly of bacteria belonging to the genera *Pseudomonas*, *Acinetobacter*, *Aeromonas* and *Stenotrophomonas* and to the families Oxalobacteraceae, Enterobacteriaceae and Comamonadaceae. We showed that both dietary regime and age were associated with the abundance of some bacterial groups in the *Ae. aegypti* microbiota. The majority of the bacterial groups we identified have been detected in the midgut of *Ae. aegypti* from laboratory and wild populations, indicating a possible core microbiota associated with this mosquito species. Our findings suggest that *Ae. aegypti* harbours a stable bacterial community during its adult life, similar to mosquito populations from distinct geographic areas, which may be further explored for arbovirus biocontrol strategies.

The microbial communities colonising cells and organs of higher organisms (microbiota) interact with hosts in a continuum of ecological interactions ranging from parasitism to mutualism. The microbiota is believed to provide the host with valuable capabilities, which influence physiology and improve fitness (Rosenberg & Rosenberg 2008). The insect digestive tract harbours microbial communities, comprised of viruses, bacteria, yeasts and protists. Insect vector bacterial microbiota, especially of mosquitoes, is known to play an important role in host nutrition, reproduction, development, immunity and protection against pathogens (Minard et al. 2013).

Recent reports have demonstrated that mosquito midgut microbiota is a key component of vector competence, as gut bacteria can inhibit the development of pathogens, including *Plasmodium* and dengue virus (DENV) (Xi et al. 2008, Dong et al. 2009, Ramírez et al. 2012, 2014). These findings suggest the introduction of particular bacterial strains, harbouring innate or engineered anti-pathogen activity, into the mosquito gut as a novel disease control strategy (Gusmão et al. 2010, Ramírez et al. 2012, 2014, Villegas & Pimenta 2014). For the effective application of this approach in the field, it is mandatory to know which factors influence the microbiota diversity of the target vector species, especially under natural conditions.

In the laboratory environment, dietary regime (Gusmão et al. 2010, Terenius et al. 2012) and chikungunya virus infection (Zouache et al. 2012) influenced the microbiota diversity of adult *Aedes* mosquitoes. In wild populations, the host genetic diversity (Minard et al. 2015) and habitat (Zouache et al. 2011, Osei-Poku et al. 2012, Kim et al. 2015) modulated the midgut community composition. In this scenario, the understanding of midgut bacterial community dynamics throughout the *Aedes aegypti* lifespan, under natural conditions, is still imperative.

The objective of this study was to profile the midgut microbial diversity throughout the *Ae. aegypti* lifespan and address possible determinants of microbiota structure in adult females. Since the environment is believed to influence midgut community composition in mosquitoes (Zouache et al. 2011, Osei-Poku et al. 2012, Kim et al. 2015), we investigated whether the release of laboratory reared females into the urban environment would result in changes in their microbiota composition over time. Therefore, we performed a mark-release-recapture experiment, a reliable tool for the ecological study of a target species in its natural habitat. We identified gut bacteria employing culture-independent (deep-sequencing) and -dependent approaches in female mosquitoes recaptured on different days after release. We also characterised the microbiota diversity according to diet (sugar or blood) and mosquito age in laboratory and wild insect populations.

## MATERIALS AND METHODS

*Study area* - The study was conducted in a section of 15 blocks in Vila Valqueire, a middle class suburban district located in the northeast of Rio de Janeiro city, RJ, Brazil (22º53′09′′S; 43º21′59′′W). It is a sparsely populated area surrounded by a secondary forest with paved streets, regular municipal water supply and garbage collection. The region contains 410 houses, a majority with three-four bedrooms and extended yards, with human density of 7,4 inhabitants/km^2^. This neighbourhood was chosen for the mark-release-recapture experiment because it offered the highest mosquito collection rate, among the five locations in Rio de Janeiro monitored by our group, with dengue outbreaks occurring every three-four years. Weekly sampling in Vila Valqueire for two consecutive years revealed an average of 1.3 female mosquitoes per house per day. Water tanks and buckets have been indicated as the most productive *Ae. aegypti* breeding containers (Dutra et al. 2015).

*Mark-release-recapture experiment* - The *Ae. aegypti* mosquitoes (F1 generation) were derived from a laboratory colony established from eggs collected in Vila Valqueire with 40 ovitraps uniformly distributed throughout the neighbourhood, to collect a representative sample of the genetic variation in the population. Larvae for the experiment were fed commercial fish food (Tetramin^®^, Spectrum Brands, Madison, WI, USA). Adults were fed *ad libitum* 10% sucrose, until the release day. Males and females were maintained in the same cage for 72 h under laboratory conditions (25 ± 5ºC and 60 ± 8% relative humidity) to stimulate mating.

Mated and sugar-fed three-day-old females were marked with fluorescent dust (Day-Glo Color Corp., Cleveland, OH, USA) and then released outdoors at a local square, to ensure mosquito dispersal through the neighbourhood. Daily captures were performed with backpack aspirators (John W Hock, Gainesville, FL, USA), starting on the day following release. A total of 15 houses were randomly selected daily for insect aspiration for 15-20 min per house, including the peridomestic area. Mosquitoes were recaptured within a range of 0.5 km, according to the short flight range usually observed for *Ae. aegypti* (McDonald 1977, Harrington et al. 2005, Maciel-de-Freitas et al. 2007).

The recaptured females were transported alive to the laboratory to preserve DNA quality, anaesthetised on ice, and checked for the fluorescent marking under UV light. Captures ended when no marked females were collected in three consecutive days. Fiocruz Ethical Committee approved the mark-release-recapture experiment in Vila Valqueire (CEP protocol number 22286313.7.0000.5248); permission was also obtained from the Rio de Janeiro Department of Health. In order to recapture marked mosquitoes inside dwellings, local residents received a full explanation of the project from at least one of the co-authors. In addition, mosquito collection is vector surveillance routine in Rio de Janeiro; therefore, many residents are used to receiving health agents in their houses. Collections were performed after obtaining formal written consents from the householders. There were no releases during dengue outbreaks or if dengue cases were reported in that neighbourhood.

*Midgut microbiota samples* - The diversity of midgut microbiota of *Ae. aegypti* females collected from the field was evaluated by 16s rDNA gene pyrosequencing in (a) female mosquitoes released and recaptured (MRR) two, four, six, seven and eight days after release (DAR) and (b) wild females (WD). The profiling of wild insects, i.e. unmarked insects, was conducted to provide control for the effect of laboratory rearing on female gut microbiota. The majority of females recaptured during the mark-release-recapture assay (81%) had blood content in the midgut, a condition for microbiota investigation. Bacterial isolation was performed for all groups, except WD (Table I). The likelihood of collecting naturally infected mosquitoes during an interepidemic period is low; therefore, we did not conduct any assay to test whether the captured mosquitoes were infected with DENV.

**TABLE I:** Characterisation of *Aedes aegypti* microbiota groups selected for diversity investigation, via 16S rRNA gene pyrosequencing and bacterial isolation

Group (abbreviation)	Age (days)	Diet	N samples	Mosquitoes analysed	Sample IDs
Sugar-fed young (SFY)	3	Sugar	4	8	SFY1, SFY2, SFY3, SFY4
Blood-fed young (BFY)	3	Sugar + Blood	2	4	BFY1, BFY2
Sugar-fed old (SFO)	38	Sugar	4	8	SFO1, SFO2, SFO3, SFO4
Mark-release-recaptured (MRR)*	5 to 11	Sugar + Blood	8	15	TwoDAR1, TwoDAR2, FourDAR1, FourDAR2, SixDAR1, SixDAR2, SevenDAR, EightDAR
Wild (WD)**	Unknown	Unknown	4	8	WD1, WD2, WD3, WD4

*: samples from two, four, six, seven and eight days after release (DAR). Each sample consisted of two pooled midguts with the exception of one sample from MRR (EightDAR) in which only one female was available. Bacteria were isolated from samples four, seven and eight DAR; **: bacterial isolation not performed.

A portion of the mosquitoes from the same generation/batch not released in the natural environment was maintained in the laboratory, where we assessed the microbiota of (c) sugar-fed young (SFY), (d) blood-fed young (BFY), and (e) sugar-fed old (SFO) females (Table I). This aided in determining the effect of diet regime on microbiota composition before and after blood feeding. In addition, we seldom recaptured females ~10 days post release; therefore, we also sampled 38-day-old laboratory insects to evaluate the effect of ageing on microbiota diversity.

*Midgut dissection* - Only live insects were processed in order to preserve DNA quality for microbiota investigation. Ice-anaesthetised mosquitoes were surface-sterilised in 70% ethanol for 1 min and rinsed with sterile phosphate-buffered saline (PBS). Soon after surface sterilisation, mosquitoes were submerged in sterile PBS. An aliquot of this PBS was plated on Luria-Bertani agar media (LBA), which was kept at room temperature for seven days. This procedure was conducted to ensure mosquito external surface sterilisation. If bacterial growth occurred on the LBA, the corresponding samples were discarded, because they were potentially contaminated by bacteria from the external surface of the mosquitoes. Midguts were removed on a sterile glass slide and macerated in sterile PBS. Each sample consisted of two midguts. Females from the mark-release-recapture experiment were coupled according to their recapture day and the presence of blood in their intestinal tract.

*16S rRNA library construction and 454 pyrosequencing* - Total DNA from the midgut samples was extracted with the DNeasy Blood & Tissue Kit (Qiagen, Redwood City, CA, USA). The hypervariable regions (V3 to V5) of bacterial 16S rRNA gene were amplified with 926R (5′-CCGTCAATTCMTTTRAGT-3′) and 357F (5′-CCTACGGGAGGCAGCAG-3′) primers containing 454 sequencing adapters and Multiplex Identifier (MID) tags, affording the multiplexing of samples (HMP Consortium, 2010). Polymerase chain reaction (PCR) was performed with Platinum Taq DNA Polymerase High Fidelity (Invitrogen, Carlsbad, CA, USA), with initial denaturation at 95ºC/2 min and 30 cycles of denaturation at 95ºC/20 sec, annealing at 50ºC/30 sec, and extension at 72ºC/5 min (HMP Consortium, 2010). Each 16S rRNA amplicon library was constructed from three independent PCRs. PCR products were purified with the Agencourt AMPure XP kit (Beckman Coulter, Brea, CA, USA). Twenty-four libraries were constructed, 22 from midgut samples and two controls. Pyrosequencing was performed using a high-throughput platform/Fiocruz, with the 454 Genome Sequencer Junior System (Roche, Basel, Switzerland). Sequences were deposited in the National Center for Biotechnology Information Sequence Reads Archive (accession number SRR2916651).

*Bioinformatics and operational taxonomic unit (OTU) assignment* - Data were trimmed, filtered and analysed with the Mothur package v.1.31.2 (Schloss et al. 2009). Sequence reads were sorted into appropriate files according to MID tags, allowing two errors on the primer sequence and one on the barcode sequence. Additionally, sequences were trimmed to 450 flowgrams. After the trimming process, reads less than 200 bp were discarded. Finally, potential chimeric sequences were removed with UCHIME, implemented on Mothur software (Edgar et al. 2011). All unique sequences were aligned to the SILVA-database reference alignment (release 119) and classified with the Mothur implementation of the naïve Bayesian classifier (Wang et al. 2007). Taxonomic assignment was based on the Ribosomal Data Project reference files (Training Set v.9) with an 80% bootstrap cut off. Pyrosequencing produced ~360,000 sequence reads from *Ae. aegypti* midgut samples. After primer and barcode removal, quality trimming, size filtering and chimera exclusion, 245,647 reads (241-307 bp long) were considered to be of good quality for taxonomic analysis.

Sequence reads with maximum 3% distance were clustered into OTUs. The OTUs represented by a single read or detected in control samples in abundance smaller than 10 times greater than in controls were discarded (Minard et al. 2015). Sample coverage was evaluated through rarefaction curves, which describe the number of OTUs detected as a function of sequencing generated reads. The sampling effort across samples was standardised by randomly selecting the smallest number of sequences among all the samples.

*Diversity and statistical analysis* - Alpha diversity was evaluated with OTU clustering through richness, abundance-based coverage estimator (ACE), Shannon index, inverse Simpson index and evenness. Diversity metrics were compared between groups with one-way analysis of variance (ANOVA; followed by Tukey’s post-hoc test) or Kruskal-Wallis test (followed by Dunn’s test), depending on normal distribution verification with the Shapiro-Wilk test. P-values were adjusted for multiple comparisons by the Bonferroni criteria.

Principal coordinates analysis (PCoA) and non-parametric multivariate ANOVA (NPMANOVA or “adonis”), both with a Bray-Curtis dissimilarity matrix, were employed to assess the overall dissimilarity in the community structure over the groups of samples (Table I). NPMANOVA tests, for the difference between means or centroids, were performed by comparing the variability within groups versus the variability among groups, for data presenting many response variables (i.e. OTUs at 3% distance) without assuming a multivariate normal distribution (Anderson 2001). Diversity metrics calculation, ordination and statistical analysis were performed with the R ‘vegan’ package (Oksanen et al. 2013).

Microbiota composition among the groups of *Ae. aegypti* females was compared by performing (a) a neighbor-joining tree with representative 16S rRNA gene sequences of bacterial taxa (families or genera), coupled with presence/absence data (Letunic & Bork 2011), (b) a Venn diagram, and (c) an abundance statistical comparison between taxa exhibiting > 2% relative abundance using the Kruskal-Wallis test, followed by pairwise comparisons with the Dunn’s test, if necessary. P-values were adjusted by controlling the false discovery rate (FDR) using the Benjamini-Hochberg procedure. Additionally, relative abundance was also compared among sugar-fed (SFY, SFO) and blood-fed samples (BFY, MRR) (Table I). P-values were adjusted by the Bonferroni method. We used a level of significance for corrected p-values < 0.05. All statistical analyses were performed in R environment (R Development Core Team 2015).

*Prediction of potential microbial function* - In order to assess whether the diversity changes were associated with differences in the functional profile of *Ae. aegypti* female microbiota, the functional composition of the gut community was predicted with Phylogenetic Investigation of Communities by Reconstruction of Unobserved States (PICRUSt) (Langille et al. 2013) on the online Galaxy interface. Briefly, this bioinformatics approach incorporates a marker gene (16S rRNA gene) and a database of reference genomes to infer, by ancestral state reconstruction, the functional profile of host-associated and environmental communities (Langille et al. 2013). The nearest sequenced taxon index (NSTI) indicates the accuracy of metagenome predictions, accuracy increasing with decreasing NSTI. Our samples had NSTI of 0.02 ± 0.002, considered tractable for PICRUSt prediction (Langille et al. 2013). The predicted genes were annotated using the Kyoto Encyclopedia of Genes and Genomes (KEGG) database. Level 2 KEGG Orthology groups, with more than 2% relative abundance, were compared by the Kruskal-Wallis test followed by pairwise comparisons with the Dunn’s test, if necessary, in R environment (R Development Core Team 2015). P-values were adjusted by controlling the FDR using the Benjamini-Hochberg procedure.

*Isolation of midgut bacteria* - Bacterial isolation was conducted with the aim to provide an overview of the culturable microbiota of the Vila Valqueire *Ae. aegypti* population. We selected four samples from each group, except from BFY in which only two samples were available. For the MRR samples, isolation was conducted after three-four days of collection. Isolation was not performed in wild insects due to logistic issues. Soon after the dissection, each sample was diluted 10-fold and plated on LBA agar and 10-fold diluted tryptone soya agar (TSA). For the ensuing 72 h, bacterial colonies were screened based on morphology and samples of each morphotype were cryopreserved.

Bacterial DNA was extracted by a boiling and freezing procedure. A 16S rRNA gene segment (~519 bp), between the V1 and V3 hypervariable regions, was amplified using the 16S universal primers (forward 5′-AGAGTTTGATCCTGGCTCAG-3′ and reverse 5′-GTATTACCGCGGCTGCTG-3′). This segment allows the identification of a majority of bacterial groups at genus level. For families with highly similar 16S rRNA gene sequence (e.g., Enterobacteriaceae), a larger fragment (~1,000 bp) between the V1 and V5 hypervariable gene regions was sequenced (forward 5′-AGAGTTTGATCCTGGCTCAG-3′ and reverse 5′-GTTGCGCTCGTTGCGGGACT-3′). PCR was performed with GoTaq Flexi DNA Polymerase (Promega, Madison, WI, USA) under standard conditions. Amplicons were purified with the Illustra PCR and Gel Band Purification Kit (GE Healthcare, Little Chalfont, BUX, UK) and sequenced using BigDye Terminator v3.1 Cycle Sequencing Kit, in an ABI 3730 DNA sequencer (Applied Biosystems, Foster City, CA, USA).

Bacteria were identified with the Ribosomal Data Project classifier program (https://rdp.cme.msu.edu/classifier, Version 2.10), with a confidence threshold of 80% at the genus level. All sequences were submitted to GenBank. A neighbor-joining tree was constructed with a representative 16S rRNA gene sequence for each bacterial genus, coupled with presence/absence data (Letunic & Bork 2011).

## RESULTS

The midgut microbial diversity throughout *Ae. aegypti* lifespan was investigated after the release of laboratory reared females into the urban environment. The microbiota of recaptured specimens was contrasted with the microbiota of laboratory females on different diet and of different age groups, as well as with wild insects from natural breeding sites, employing deep sequencing of bacterial 16S rRNA gene and bacterial isolation (Table I).

**Fig. 1:**
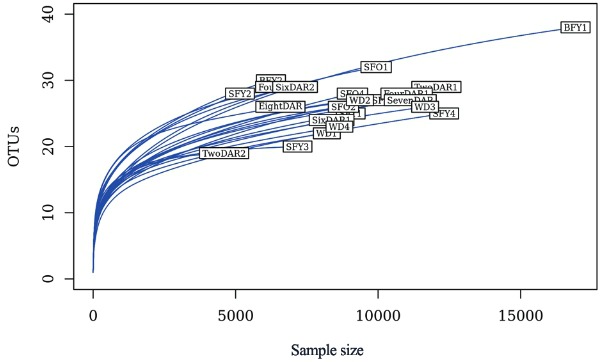
: rarefaction curves for each sample. Operational taxonomic units (OTUs) at 3% distance were plotted for each sample against the number of sequences generated through 454 pyrosequencing (Sample size). SFY: sugar-fed young; BFY: blood-fed young; SFO: sugar-fed old; DAR: days after release in the mark-release-recapture experiment; WD: wild.

*Bioinformatics processing and diversity metrics* - Sequences assigned to the bacteria domain varied, from 4,599-16,935, with conditions. Sequence reads were clustered at maximum 3% distance into 90 non-singleton OTUs. Sequencing coverage for each group was assessed through rarefaction curves. The curves levelled off without reaching a plateau, suggesting that most microbiota OTUs were detected but some possibly remaining undiscovered (Fig. 1).

As the sample size was constrained by the low recapture rate of the mark-release-recapture experiment [we released 1,730 *Ae. aegypti* and recaptured 67 (~4%) of them using backpack aspirators, with ~54% in the first two days of collection] and by the methodological procedures (only alive females were dissected), we opted to use all the samples in the analysis and standardised the sampling effort to 4,599 reads. Average richness of OTUs per group ranged from 20.75 (SFO) to 24.5 (BFY). Estimated total richness by ACE varied from 25.3 (SFO) to 33.47 (BFY) (Table II). There were no significant differences among groups for either observed or estimated richness. On the other hand, SFO mosquitoes presented significantly higher Shannon index (z = 3.48, p-value < 0.01), inverse Simpson index (z = 3.15, p-value < 0.01), and evenness (z = 3.81, p-value < 0.01) than WD mosquitoes (Table II).

**TABLE II:** Average ± standard deviation of diversity metrics calculated for the bacterial communities associated with the analysed *Aedes aegypti* groups

Group	S	ACE	H’	1/D	J’
SFY	21.25 ± 3.20	27.21 ± 6.2	1.29 ± 0.21	2.46 ± 0.51	0.42 ± 0.05
BFY	24.50 ± 2.12	33.47 ± 2.16	1.34 ± 0.17	2.38 ± 0.36	0.42 ± 0.04
SFO	20.75 ± 0.96	25.30 ± 1.89	1.58 ± 0.10	3.66 ± 0.69	0.52 ± 0.03
MRR	22.25 ± 3.92	27.55 ± 6.75	1.32 ± 0.13	2.40 ± 0.30	0.43 ± 0.03
WD	21.25 ± 1.26	29.88 ± 5.03	1.14 ± 0.05	2.10 ± 0.10	0.37 ± 0.02

S: richness (number of operational taxonomic units); ACE: abundance-based coverage estimator; H’: Shannon index; 1/D: inverse Simpson index; J’: evenness; SFY: sugar-fed young; BFY: blood-fed young; SFO: sugar-fed old; MRR: mark-released-recaptured; WD: wild.

**Fig. 2:**
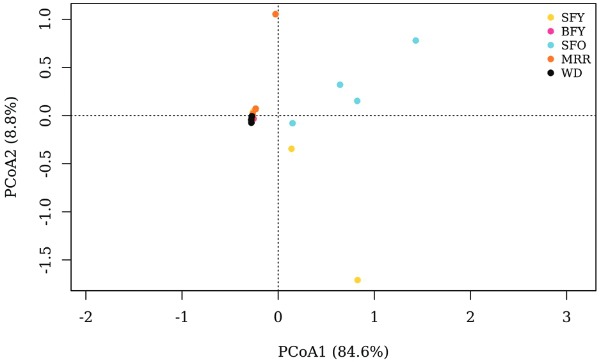
: principal coordinate analysis (PCoA) of Bray-Curtis distances among *Aedes aegypti* microbiota samples. Bray-Curtis distance was calculated with operational taxonomic units at 3% maximum distance. SFY: sugar-fed young (yellow); BFY: blood-fed young (pink); SFO: sugar-fed old (blue); MRR: mark-released-recaptured (orange); WD: wild (black). Each axis shows percentage of variation explained.

PCoA displayed a single cluster for two SFY samples, BFY, MRR (except TwoDAR1) and WD, whereas there was a higher intra- and intergroup variability in bacterial community structure between the two SFY and SFO samples (Fig. 2). Differences in microbiota community structure among groups were supported by statistical analysis (NPMANOVA: R^2^ = 0.62, p-value < 0.01).

**Fig. 3:**
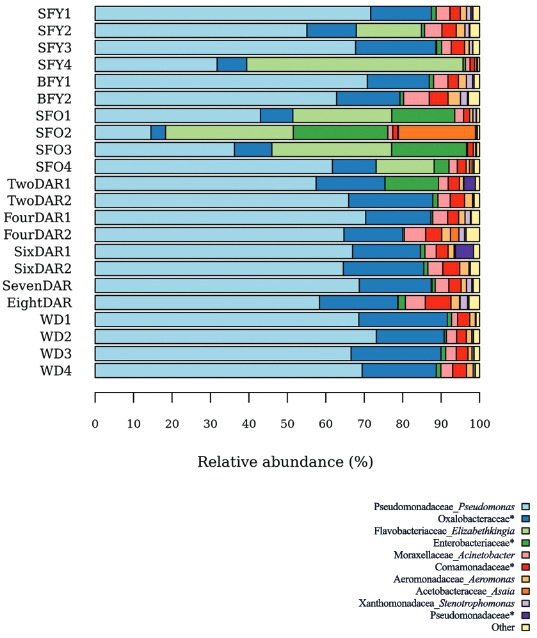
: *Aedes aegypti* microbiota composition per sample. Gut bacterial taxonomic composition of *Ae. aegypti* adult females was determined via 16S rRNA gene deep sequencing. SFY: sugar-fed young; BFY: blood-fed young; SFO: sugar-fed old; DAR: days after release in the mark-release-recapture experiment; WD: wild. Taxa with < 2% relative abundance were pooled as “Other”; *: not discriminated at genus level.

*Taxonomic assignment and microbiota composition* - In total, more than 99% and 76% of the reads were assigned to family and genus levels, respectively. Only non-singleton taxa were considered for further analysis. Four bacterial phyla, 23 families and 32 genera were successfully identified in the *Ae. aegypti* gut microbiota. The bacterial communities colonising female midguts were composed of Proteobacteria (43.7 to 99.9%), followed by Bacteroidetes (0 to 56.3%), Firmicutes (0 to 0.3%) and Actinobacteria (0 to < 0.1%). Detailed community composition per sample, at family and genus levels, is represented in Fig. 3 and Supplementary Table.

**Fig. 4:**
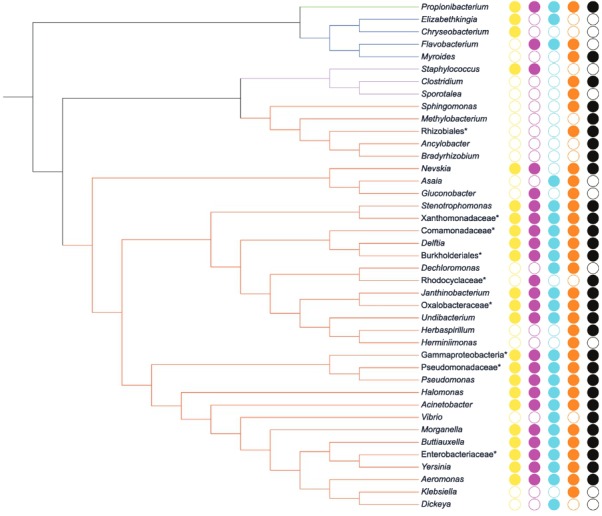
: phylogenetic tree of bacterial taxa detected in *Aedes aegypti* female midgut via 16S rRNA gene deep sequencing. Yellow circles indicate taxa identified in sugar-fed young females (SFY); pink circles in blood-fed young (BFY); light blue in sugar-fed old (SFO); orange in mark-released-recaptured (MRR); and black in wild (WD) females. Taxa were classified according to their phyla: Bacteroidetes (blue), Actinobacteria (green), Firmicutes (purple), and Proteobacteria (red); *: not discriminated at genus level.

**Fig. 5:**
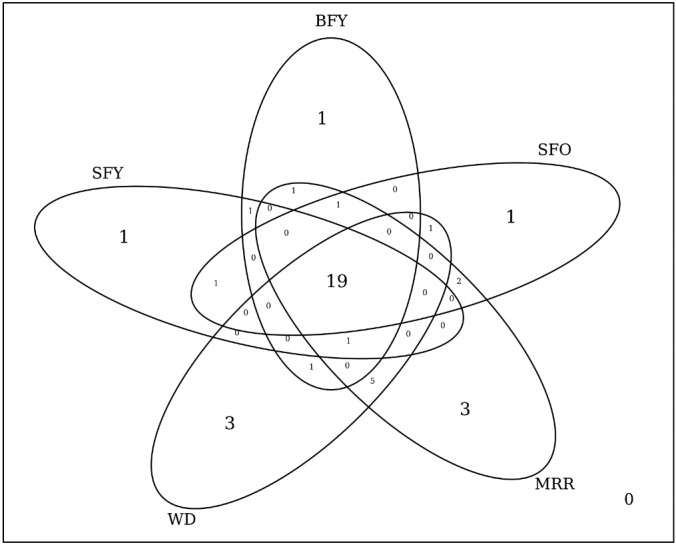
: Venn diagram showing the number of bacterial taxa identified in the *Aedes aegypti* microbiota via 16S rRNA gene deep sequencing. SFY: sugar-fed young; BFY: blood-fed young; SFO: sugar-fed old; MRR: mark-released-recaptured; WD: wild.

**Fig. 6:**
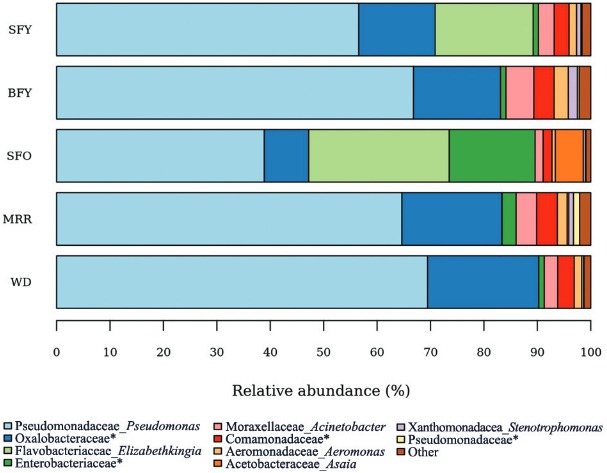
: *Aedes aegypti* microbiota composition per group. Gut bacterial taxonomic composition of *Ae. aegypti* adult females was determined via 16S rRNA gene deep sequencing. SFY: sugar-fed young; BFY: blood-fed young; SFO: sugar-fed old; MRR: mark-released-recaptured; WD: wild. Taxa with < 2% relative abundance were pooled as “Other”; *: not discriminated at genus level.

The core microbiota (taxa present in all groups) consisted of 19 out of 41 (~46%) bacterial taxa (Figs 4-5), comprising 46 to 99% of microbiota. *Pseudomonas* was the most abundant genus in the groups, composing up to 70% of the midgut bacteria in WD females (Fig. 6). The number of taxa per group was 23 in SFY, 24 in BFY, 25 in SFO, 32 in MRR, and 30 in WD mosquitoes (Fig. 4). Together, MRR and WD females contained 11 out of 41 (~26%) taxa present only in these groups, contrasting with four out of 41 taxa (~9%) exclusive to SFY, BFY and/or SFO mosquitoes collectively (Figs 4-5).

There were no significant changes in microbiota composition among MRR and BFY, WD, or SFY females, despite the higher abundance of the *Elizabethkingia* genus in two SFY samples. On the other hand, 38-day-old SFO specimens presented higher abundance of *Elizabethkingia* (vs. BFY: z = -2.44, p-value < 0.05; vs*.* MRR: z = -2.91, p-value < 0.01; vs*.* WD: z = 2.99, p-value < 0.05) and *Asaia* (vs. SFY: z = 3.01, p-value < 0.05; vs. BFY: z = -2.46, p-value < 0.05; vs*.* MRR: z = -2.58, p-value < 0.05; vs*.* WD: z = 3.01, p-value < 0.01). Furthermore, the microbiota of SFO insects displayed lower abundance of *Pseudomonas* (vs. WD: z = -2.88, p-value < 0.05), Oxalobacteraceae (vs*.* MRR: z = 2.82, p-value < 0.05; vs*.* WD: z = -3.15, p-value < 0.01), *Acinetobacter* (vs. BFY: z = 2.71, p-value < 0.05; vs*.* MRR: z = 2.89, p-value < 0.05), Comamonadaceae (vs*.* MRR: z = 2.98, p-value < 0.05), and *Aeromonas* (vs. BFY: z = 2.84, p-value < 0.05; vs*.* MRR: z = 2.84, p-value < 0.05) (Fig. 6). Strictly sugar-fed mosquitoes (SFY, SFO) exhibited significantly higher number of *Elizabethkingia* than blood-fed mosquitoes (BFY, MRR) in their microbiota (chi-squared = 10.1, degrees of freedom = 1, p-value < 0.05).

**Fig. 7:**
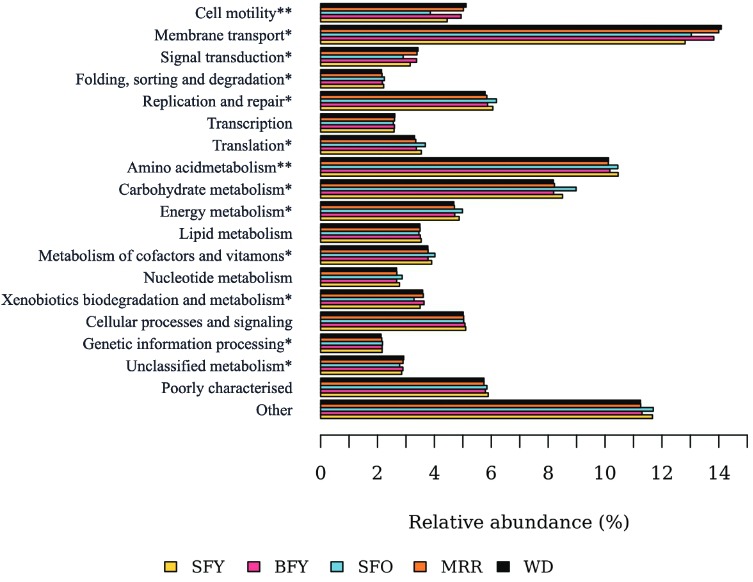
: predicted functions of the bacterial communities found in *Aedes aegypti* adult females. Yellow bars indicate Kyoto Encyclopedia of Genes and Genomes metabolic pathways predicted in the microbiota of sugar-fed young females (SFY); pink bars in blood-fed young (BFY); light blue in sugar-fed old (SFO); orange in mark-released-recaptured (MRR); and black in wild females (WD). *: indicates gene categories significantly different between SFO and WD mosquitoes; **: indicates gene categories significantly different among SFO and both MRR and WD mosquitoes (Kruskal-Wallis test, Benjamini-Hochberg corrected p-value < 0.05).

*Potential microbial function* - SFO *Ae. aegypti* demonstrated significant differences in microbiota composition when compared to BFY, MRR and WD *Ae. aegypti* females, with an increase in *Elizabethkingia* and *Asaia* and a reduction in Oxalobacteraceae, *Acinetobacter*, and *Aeromonas* bacteria. Using PICRUSt to make exploratory inferences of the microbial function, we encountered 13 gene families predicted to be differentially represented in SFO in relation to WD females (p-value < 0.05). In addition, two of these predicted functions also showed statistically significant differences between SFO and MRR females (p-value < 0.05; Fig. 7).

**Fig. 8:**
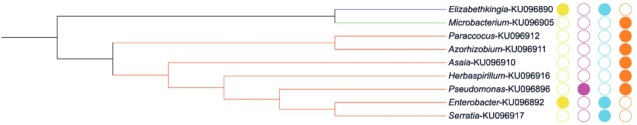
: phylogenetic tree of culturable bacterial genera recovered from *Aedes aegypti* females. SFY: sugar-fed young (yellow); BFY: blood-fed young (pink); SFO: sugar-fed old (blue); MRR: mark-released-recaptured (orange). Bacterial genera were classified according to their phyla: Bacteroidetes (blue), Actinobacteria (green) and Proteobacteria (red).

*Diversity of culturable bacteria* - The culturable midgut microbiota was represented by 39 bacterial colonies, corresponding to 24 morphotypes. Most morphotypes were isolated from both LBA and TSA media (Table III). The identification to the genus level was based on the 16S rRNA gene sequence. We recovered bacteria belonging to three phyla, eight families and nine genera (Table III, Fig. 8). The majority of genera belonged to the Proteobacteria phylum (Fig. 8). *Elizabethkingia* and *Enterobacter* were recovered from SFY group, whereas only *Pseudomonas* was isolated from BFY group. SFO females still carried *Elizabethkingia* and *Enterobacter* 35 days later, but *Serratia* was also detected in the tested specimens. An expressive change was observed in the culturable microbiota composition after release, as MRR females presented *Asaia*, *Azorhizobium*, *Microbacterium*, *Pseudomonas*, *Paracoccus* (FourDAR) and *Herbaspirillum* (EightDAR), whereas *Enterobacter* and *Elizabethkingia* were no longer cultivable. *Pseudomonas* was the only genus shared between blood-fed mosquitoes, from both MRR and laboratory (BFY) (Table III, Fig. 8).

**TABLE III:** Taxonomic affiliation of strains isolated from *Aedes aegypti* females released in the natural habitat (MRR) and submitted to different dietary regimes in the laboratory (SFY, BFY and SFO)

Phylum	Class	Family	Presumable genera (RDP classifier)	Genbank accession numbers	Isolation media	Source
Bacteroidetes	Flavobacteria	Flavobacteriaceae	*Elizabethkingia*	KU096882 - KU096890	LB, TSA 0.1x	SFY; SFO
Proteobacteria	Gammaproteobacteria	Enterobacteriaceae	*Enterobacter*	KU096891 - KU096894	LB, TSA 0.1x	SFY; SFO
Proteobacteria	Gammaproteobacteria	Pseudomonadaceae	*Pseudomonas*	KU096895 - KU096904	LB, TSA 0.1x	BFY; MRR (FourDAR)
Actinobacteria	Actinobacteria	Microbacteriaceae	*Microbacterium*	KU096905 - KU096906	LB, TSA 0.1x	MRR (FourDAR)
Proteobacteria	Alphaproteobacteria	Acetobacteraceae	*Asaia*	KU096907 - KU096910	LB, TSA 0.1x	MRR (FourDAR)
Proteobacteria	Alphaproteobacteria	Xanthobacteraceae	*Azorhizobium*	KU096911	LB	MRR (FourDAR)
Proteobacteria	Alphaproteobacteria	Rhodobacteraceae	*Paracoccus*	KU096912 - KU096915	LB, TSA 0.1x	MRR (FourDAR)
Proteobacteria	Betaproteobacteria	Oxalobacteraceae	*Herbaspirillum*	KU096916	LB	MRR (EightDAR)
Proteobacteria	Gammaproteobacteria	Enterobacteriaceae	*Serratia*	KU096917	LB	SFO

SFY: sugar-fed young; BFY: blood-fed young; SFO: sugar-fed old; DAR: days after release in the mark-release-recapture experiment; MRR: mark-released-recaptured.

## DISCUSSION

The midgut microbiota of mosquitoes is supposedly a function of host intrinsic factors (e.g., genetics), feeding behaviour and environment (e.g., climate, larval habitats and vertebrate hosts) (Zouache et al. 2011, Osei-Poku et al. 2012, Hegde et al. 2015). Nevertheless, gut community ecology remain poorly investigated from the standpoint of mosquito interactions within its natural environment. Therefore, the present study assessed the diversity and the dynamics of *Ae. aegypti* microbiota under natural conditions, using the mark-release-recapture experiment. Herein, we offer evidence for *Ae. aegypti* female lifelong core microbiota, i.e. the community composition comprising the majority of microbiota was stable after mosquitoes were released into the urban settlement. Additionally, we show that the female dietary regime and mosquito age were associated with the abundance of some bacterial groups in the *Ae. aegypti* microbiota*.*

After release, the *Ae. aegypti* females presumably blood-fed on a range of vertebrate hosts with diet supplements of different carbohydrate sources (fruits and flowers), which could represent potential sources of bacteria acquisition during their adult life. In contrast to the blood meals, the sugar meals do not go directly to the midgut, but are stored in the crop as food reserve (Clements 1992). This structure harbours bacteria, such as *Bacillus*, *Serratia* and *Pichia* (Gusmão et al. 2007), which have also been detected in the midgut (Gusmão et al. 2010). Therefore, it is suggested that the bacteria acquired from sugar sources can be transferred to the midgut along with food, when convenient (Gusmão et al. 2007).

For > 2% abundant bacteria, MRR female microbiota composition was not different from the specimens maintained in the laboratory under SFY or BFY regimes, despite of two SFY samples presenting high levels of the *Elizabethkingia* genus. In addition, microbiota was similar between laboratory reared (SFY, BFY and MRR) and WD mosquitoes, regardless of the differences between conditions in laboratory and natural breeding sites, i.e. water biogeochemical characteristics, microclimate and food type. This relatively constant midgut community composition in adult mosquitoes may reflect a competitive environment (Terenius et al. 2012) and is of great significance for the application of midgut microbiota in disease control approaches, as association of bacteria with host must be steady over the host adult lifetime (Capone et al. 2013).

MRR females exclusively shared < 2% abundant bacteria taxa with WD insects suggesting bacterial acquisition in the natural habitat after release. SFO females, which remained in the laboratory for 35 days, displayed fewer differences in microbiota composition in relation to SFY and BFY mosquitoes, indicating that this apparent midgut bacterial acquisition did not take place at the same magnitude within the laboratory environment. In general, MRR female microbiota was structurally more similar to WD and BFY than to SFY and SFO midgut communities (Fig. 2), suggesting that the environment and the feeding behaviour can synergistically modulate the presence of some bacterial groups.

Sugar-fed females (SFY and SFO) harboured the *Elizabethkingia* (Flavobacteriaceae) genus, which was absent or undetectable in blood-fed (BFY and MRR) and WD specimens. A reduction in *Elizabethkingia* was observed in *Anopheles gambiae* after blood feeding, applying the same deep sequencing approach (Wang et al. 2011). We recovered *Serratia* from sugar-fed (SFO) females, whereas a previous study reported this genus was dominant in the *Ae. aegypti* midgut after sugar feeding and during the whole blood digestion (Gusmão et al. 2010). *Asaia* was previously isolated from sugar-fed *Ae. aegypti* and upon initial blood digestion (Gusmão et al. 2010), whereas we recovered this genus in blood-fed MRR mosquitoes four days after release. Thus, strains isolated from distinct mosquito populations might differ in their growth characteristics inside the midgut environment and/or in the isolation media.

Contrasting the stability of microbiota composition in the first week after adult emergence, the microbiota of SFO females revealed a remarkable increase in *Elizabethkingia* and, to a lesser extent, *Asaia* (Acetobacteraceae). In addition, significant functional and metabolic alterations were predicted in the microbiota of SFO versus WD and MRR females. Microbial community shifts over time could be related to the mosquito immune capacity to control proliferation of certain bacteria (Hillyer et al. 2005), nutrient availability in the gut environment (Montagna et al. 2015) and/or result of direct interaction among colonising bacteria, *e.g.* competition (Dong et al. 2009).

The 16S rRNA gene sequences (1,020 bp) from *Elizabethkingia* strains recovered in this study share 99% identity with the Che01 strain (GenBank accession JX067927) isolated from the midgut of *Anopheles stephensi.* The ethyl acetate and acetone extracts produced by the Che01 strain showed antibacterial, antifungal and antiplasmodial activities, which might explain the dominance of this bacteria in the microbiota of *An. stephensi* (Ngwa et al. 2013). Similarly, *Asaia* have been shown to inhibit the colonisation and maternal transmission of the intracellular bacteria *Wolbachia* (Hughes et al. 2014). Further investigation is required to determine whether the *Ae. aegypti* microbiota strains possess similar capabilities.

Studies on the microbiota of *Ae. aegypti* females recovered *Asaia*, *Elizabethkingia, Enterobacter*, *Pseudomonas* and *Serratia* genera from laboratory colonies (Gusmão et al. 2010, Terenius et al. 2012) as well as wild populations from Panama (Ramírez et al. 2012). Additionally, 50% of the genera detected through deep sequencing were also found in *Ae. aegypti* from Kenya, applying the same approach (Osei-Poku et al. 2012). The overlapping microbiota composition among *Ae. aegypti* populations from distinct geographic regions suggest a core microbiota stably associated with this species, acquired through mechanisms of vertical transmission and/or constant environmental exposure (Zouache et al. 2011). *Ae. aegypti* breeds almost exclusively in domestic containers recurrent across the globe, such as discarded tires and plastic buckets, in which several of this bacteria taxa have been detected (Ponnusamy et al. 2008, Dada et al. 2014). This could partially explain the similarities found in the microbiota of geographically distant populations.

The present study shows that (i) the dominant bacteria were ubiquitous in the female mosquito midgut during the first week after emergence; this core microbiota was independent of rearing conditions (laboratory or natural breeding site) and diet regime (sugar or blood) after being released into the natural habitat, where females fed on blood and carbohydrate sources; (ii) the *Elizabethkingia* genera was associated with sugar-fed females; (iii) the microbiota structure changed in older mosquitoes; (iv) the shifts in the microbiota of older *Ae. aegypti* females were predicted to influence the functional and metabolic profile of the midgut community; and (v) the microbiota identified during this study shared many bacterial taxa with *Ae. aegypti* from distinct geographic locations, suggesting a core microbiota associated with this mosquito species. Investigation of the factors influencing microbiota dynamic in *Ae. aegypti* provides insight into the impacts of bacterial community on mosquito biology and evolution, which can be explored for the development of new disease control tools.
