# Supplementary material for: Effects of environment, dietary regime and ageing on the dengue vector microbiota: evidence of a core microbiota throughout Aedes aegypti lifespan
Source: Mem Inst Oswaldo Cruz. 2016 Aug 25;111(9):577–87. doi: 10.1590/0074-02760160238 (PMC5027870; doi:10.1590/0074-02760160238)
Supplement: Supplementary file 1 [file 0074-0276-mioc-0074-02760160238-suppl01.pdf]

Mem Inst Oswaldo Cruz, Rio de Janeiro, Vol. *111*(9): 1-1, September 2016

SUPPLEMENTARY TABLE

Aedes aegypti microbiota composition (number of reads) per sample

Phyla	Class	Order	Family	Genus	SFY_1	SFY_2	SFY_3	SFY_4	BFY_1	BFY_2	SFO_1	SFO_2	SFO_3	SFO_4	Two-DAR_1	Two-DAR_2	Four-DAR_1	Four-DAR_2	Six-DAR_1	Six-DAR_2	Seven-DAR	Eight-DAR	WD_1	WD_2	WD_3	WD_4
Bacteroidetes	Flavobacteria	Flavobacteriales	Flavobacteriaceae	*Elizabethkingia*	0	788	13	2631	0	0	1174	1560	1423	701	0	0	0	0	0	0	7	4	0	0	0	0
Proteobacteria	Alphaproteobacteria	Rhodospirillales	Acetobacteraceae	*Asaia*	0	0	0	0	0	0	2	941	10	39	0	0	0	87	0	0	0	5	0	0	0	0
Proteobacteria	Betaproteobacteria	Burkholderiales	Comamonadaceae	*	112	162	162	43	109	228	64	58	60	96	129	171	133	194	128	193	162	329	123	111	150	152
Proteobacteria	Betaproteobacteria	Burkholderiales	Oxalobacteraceae	*	728	587	914	358	766	763	402	160	474	564	853	1006	786	679	803	965	878	913	1114	812	1061	897
Proteobacteria	Gammaproteobacteria	Aeromonadales	Aeromonadaceae	*Aeromonas*	87	105	54	28	95	154	40	7	27	44	53	96	92	112	66	116	66	122	72	55	57	63
Proteobacteria	Gammaproteobacteria	Enterobacteriales	Enterobacteriaceae	*	67	35	60	19	36	34	745	1124	912	166	675	61	26	22	47	41	34	88	53	24	45	58
Proteobacteria	Gammaproteobacteria	Pseudomonadales	Pseudomonadaceae	*Pseudomonas*	3289	2524	3148	1434	3260	2869	1980	646	1639	2777	2598	3034	3215	3007	3126	2963	3130	2664	3114	3386	3050	3185
Proteobacteria	Gammaproteobacteria	Pseudomonadales	Moraxellaceae	*Acinetobacter*	156	217	119	51	178	309	109	55	17	115	105	148	173	251	121	182	155	250	67	118	146	148
Proteobacteria	Gammaproteobacteria	Pseudomonadales	Pseudomonadaceae	*	19	9	4	1	15	18	8	4	2	4	137	8	8	21	209	8	18	16	5	4	2	4
Proteobacteria	Gammaproteobacteria	Xanthomonadales	Xanthomonadaceae	*Stenotrophomonas*	68	51	46	14	85	88	39	18	4	21	2	10	68	71	14	13	74	85	2	18	16	24
Other	Other	Other	Other	Other	73	121	79	20	55	136	36	26	31	72	47	65	98	155	85	118	75	123	49	71	72	68

SFY: sugar-fed young; BFY: blood-fed young; SFO: sugar-fed old; DAR: days after release in the mark-release-recapture experiment; WD: wild; Taxa with < 2% relative abundance were pooled as “Other”; *: not discriminated at genus level.
